# Tissue Hypoperfusion, Hypercoagulopathy, and Kidney and Liver Dysfunction after Ingestion of a Naphazoline-Containing Antiseptic

**DOI:** 10.1155/2017/3968045

**Published:** 2017-05-07

**Authors:** Yuko Ono, Nozomi Ono, Kazuaki Shinohara

**Affiliations:** ^1^Emergency and Critical Care Medical Center, Fukushima Medical University Hospital, 1 Hikarigaoka, Fukushima 960-1295, Japan; ^2^Department of Anesthesiology, Ohta General Hospital Foundation, Ohta Nishinouchi Hospital, 2-5-20 Nishinouchi, Koriyama, Fukushima 963-8558, Japan; ^3^Department of Psychiatry, Hoshigaoka Hospital, 7 Kitasanten, Katahira, Koriyama, Fukushima 963-0211, Japan

## Abstract

Naphazoline is a peripheral *α*_2_-adrenergic receptor agonist commonly used as a topical decongestant. In Japan, over-the-counter antiseptics often contain naphazoline to effect local hemostasis. We present the first case involving the development of hypercoagulopathy, with kidney and liver dysfunction, following a naphazoline overdose. A 22-year-old Japanese woman with a history of depression ingested 160 mL of a commercially available antiseptic containing 0.1% naphazoline. Three days later, she was brought to the emergency department because of general fatigue, nausea, and vomiting. Physical examination revealed cool, pale extremities. Laboratory data showed evidence of severe kidney and liver dysfunction (creatinine, 9.2 mg/dL; alanine aminotransferase, 2948 IU/L), hypercoagulation (D-dimers, 58.3 *μ*g/mL), and thrombocytopenia (platelet count, 90,000/*μ*L). After infusion of normal saline, intravenous administration of alprostadil, and hemodiafiltration, her organ function completely recovered. Because both the kidney and liver express *α*_2_-adrenergic receptors, their failure was likely associated with naphazoline overdose-induced hypoperfusion. The most plausible causes of hypercoagulation are peripheral low perfusion and subsequent microthrombus formation. This case illustrates that severe organ dysfunction can occur following over-the-counter antiseptic ingestion and serves as a caution for both drug manufacturers and healthcare professionals.

## 1. Introduction

Naphazoline is commonly used as a topical nasal and ocular decongestant, and its therapeutic effect results from stimulation of peripheral postsynaptic *α*_2_-adrenergic receptors. Similar to dexmedetomidine and clonidine, naphazoline also acts as an *α*_2_-adrenergic receptor agonist in the central nervous system. As summarized in Table 1, the most common symptoms of naphazoline overdose include consciousness disturbances, bradycardia, bradypnea, transitory arterial hypertension, miosis, and diaphoresis [1–11]. Even severe hypertension, afterload mismatch, and pulmonary edema can result from ingestion of a copious amount of naphazoline [3, 8–10]. In Japan, over-the-counter antiseptics, which often contain naphazoline to effect local hemostasis, are widely available.

Liver and kidney dysfunction following naphazoline overdose is rare [10, 11], and the etiology of naphazoline-induced organ disorders is unclear. Moreover, no previous report has described the development of hypercoagulopathy after naphazoline ingestion.

We describe a patient who developed hypercoagulopathy with kidney and liver dysfunction following ingestion of a naphazoline-containing antiseptic. The patient's symptoms were likely associated with systemic vasoconstriction and low organ and peripheral perfusion arising from the pharmacological properties of an *α*_2_-adrenergic receptor agonist.

## 2. Case Presentation

A 22-year-old Japanese woman with a 2-year history of depression attempted suicide by ingesting 160 mL of a commercially available antiseptic containing 0.1% naphazoline, 0.1% benzalkonium chloride, 0.1% chlorpheniramine, and 1.0% lidocaine. Three days later, she was brought to the emergency department (ED) because of general fatigue, nausea, and vomiting. According to her family, she had been able to take in food and water during the days before admission. Her initial vital signs recorded in the ED were as follows: body temperature, 37.3°C; heart rate, 54 beats per min; blood pressure, 109/87 mmHg; and respiratory rate, 20 breaths per min. She was oriented (Glasgow Coma Scale score of 15) but slightly agitated. Her peripheral extremities were cold, dry, and pale, and her capillary refill time was prolonged (>4 s). Her medications at the time of admission were duloxetine, aripiprazole, lorazepam, brotizolam, and flunitrazepam. She denied overdose of these medications or illicit drugs.

Laboratory data on admission revealed severe kidney and liver dysfunction (blood urea nitrogen, 77 mg/dL; creatinine, 9.2 mg/dL; alanine aminotransferase, 2948 IU/L; aspartate aminotransferase, 265 IU/L), hypercoagulation (D-dimers, 58.3 *μ*g/mL; fibrin degradation products, 98.0 *μ*g/mL), prolongation of prothrombin time (percentage of standard value, 65.3%), and thrombocytopenia (platelet count, 90,000/*μ*L). Plasma fibrinogen (180 mg/mL) and serum albumin (2.9 g/dL) levels were at the lower limit of the normal ranges, and creatine kinase (80 IU/L) and total bilirubin (0.9 mg/dL) concentrations were within normal limits. There was no evidence of hemoconcentration (hemoglobin, 11.7 g/dL; hematocrit, 34.7%; Na^+^, 135 mEq/L; and K^+^, 4.2 mEq/L). Computed tomography showed bilateral renal swelling, suggesting acute renal failure. The inferior vena cava was not collapsed on computed tomography or ultrasonography. There was no evidence of deep vein thrombosis, pulmonary thromboembolism, or crush syndrome. The patient's electrocardiogram was unremarkable other than sinus bradycardia.

The direct causation, ingestion of a naphazoline-containing antiseptic, evidence of low peripheral perfusion, and bradycardia were consistent with naphazoline intoxication. Because naphazoline is a strong agonist of peripheral *α*_2_-adrenergic receptors, hypercoagulopathy may result from peripheral vasoconstriction, hypoperfusion, and subsequent hypercoagulation and microthrombus formation. The kidney and liver failures were likely associated with systemic vasoconstriction and decreased blood flow to the organs.

Because of the patient's diaphoresis, nausea, and vomiting, the differential diagnosis of tissue hypoperfusion and kidney and liver dysfunction was dehydration. However, there was neither hemoconcentration nor inferior vena cava collapse at the time of admission. These findings made dehydration-induced organ failure less likely.

The patient was treated with a transfusion of normal saline (3000 mL/day) and a 10-*μ*g intravenous bolus of alprostadil (Palux Injection; Taisho Pharmaceutical Co., Tokyo, Japan), a synthetic variant of prostaglandin E1, daily for 7 days. On day 2, the patient remained anuric and was therefore treated with hemodiafiltration. Her urine production returned by day 3; this was followed by development of the diuretic phase and complete recovery. The hypercoagulopathy and liver dysfunction also gradually resolved.

After further psychiatric evaluation and inpatient treatment, the patient was discharged home, where she returned to her normal activities. At her outpatient follow-up visit 1 month later, kidney function and liver function were normal.

## 3. Discussion

This case involved kidney and liver dysfunction arising from overdose of an antiseptic containing an *α*_2_-adrenergic receptor agonist. The kidney and liver are especially vulnerable to prolonged low perfusion because both require high blood flow relative to organ mass. The patient's hypercoagulation is likely explained by peripheral hypoperfusion and subsequent microthrombus formation. Her liver dysfunction may also have exacerbated the hypercoagulation by impairing clearance of clotting factors.

The liver and kidney receive abundant blood flow relative to organ mass. Therefore, both of these organs may be predisposed to low perfusion arising from ingestion of a copious amount of naphazoline. In addition, microthrombi may aggravate these organs' dysfunction and vice versa. In humans, both the renal cortex and hepatic vessels express *α*_2_-adrenergic receptors [12, 13]. Talke et al. [14] reported that cardiac output and renal blood flow were markedly decreased after intravenous administration of a high dose of an *α*_2_-adrenergic agonist in sheep. These experimental findings [12–14] support the pathogenesis suspected in the present case.

However, kidney failure and liver failure are not typical of naphazoline intoxication. For example, only two reported cases involved patients who developed kidney failure following naphazoline ingestion (Table 1) [10, 11]. Also, hypercoagulopathy associated with naphazoline ingestion has not been reported. Our patient presented 72 h after taking 160 mg of naphazoline. This amount is the greatest, and this delay the longest, reported in the literature (Table 1). In other patients who developed kidney and liver dysfunction, the estimated naphazoline intake was also high (150 mg) [10, 11], and initial treatment was delayed for 48 h [11]. Dose and time dependency may therefore exist between naphazoline ingestion and the development of severe organ dysfunction. Rapid restoration of organ blood flow may be vital in the treatment of naphazoline intoxication.

In addition to naphazoline, the other agents in the antiseptic ingested by this patient were 0.1% benzalkonium chloride, 0.1% chlorpheniramine, and 1.0% lidocaine. Typical symptoms of toxicity associated with these agents do not explain the liver dysfunction, kidney failure, and hypercoagulopathy observed in the present case. Nevertheless, the anticholinergic property of chlorpheniramine might have weakened the peristalsis of the gastrointestinal tract, enhancing naphazoline absorption. Benzalkonium chloride is a cationic surfactant and may therefore have aggravated the patient's dehydration and tissue hypoperfusion. Other pharmacologic components of these agents may have synergized the toxic effects of naphazoline, leading to severe organ dysfunction.

Alprostadil has potent pharmacologic effects, including vasodilation, inhibition of platelet aggregation, and improvement of microcirculation [15, 16]. Although no prior report has described the use of intravenous alprostadil for the treatment of *α*_2_-adrenergic receptor agonist overdose, we selected this agent because prostaglandin E1 has several other benefits, including hepatic and renal cytoprotection [16–18], facilitation of liver regeneration [19], and prevention of further formation of microthrombi [20]. Prostaglandin E1 has also been found to be useful in treating patients with contrast-induced nephropathy [16, 21] and fulminant hepatitis [22, 23].

In the present case, plasma and urine concentrations of naphazoline were not measured because of our limited access to the required measurement systems and small budget. In another reported case of a 23-year-old Japanese man who ingested 150 mg of naphazoline 48 h before presentation, the plasma naphazoline concentration was 1.4 *μ*g/mL [11]. We speculate that our patient had a higher plasma concentration because she had ingested 160 mg of naphazoline.

In conclusion, we have reported a case involving a patient who developed hypercoagulation, liver dysfunction, and renal failure after ingesting a large amount of a naphazoline-containing antiseptic. The likely pathogenesis in this case was vasoconstriction, low organ perfusion, and impaired microcirculation arising from systemic *α*_2_-adrenergic receptor stimulation. This case illustrates that severe organ dysfunction can occur following over-the-counter antiseptic ingestion and serves as a caution for both drug manufacturers and healthcare professionals. This case facilitated our understanding of the pathophysiology of organ failure following naphazoline overdose.

## Figures and Tables

**Table 1:** Summary of clinical characteristics of naphazoline intoxication.

Reference	Age	Sex	Estimated naphazoline intake (mg)	Symptoms	Treatment	Time before presentation (h)	Outcome
[1]	1	F	0.2	Bradycardia, miosis hypothermia, conscious disturbance, bradypnea	Close monitor	N/R	Survival

[2]	1	M	N/R	Conscious disturbance, miosis	Close monitor	1	Survival

[2]	2	F	3	Bradypnea, conscious disturbance, miosis	Close monitor	11	Survival

[3]	1	M	4	Hypertension, prolonged awaking from general anesthesia, pulmonary edema	Mechanical ventilation	N/R	Survival

[4]	5	M	100	Bradycardia, conscious disturbance, diaphoresis, hypothermia	Diuretics	1.5	Survival

[5]	7	M	N/R	Bradycardia, conscious disturbance, convulsion, headache, miosis, nausea/vomiting	Close monitor	N/R	Survival

[6]	89	M	70	Bradycardia, bradypnea, conscious disturbance, diaphoresis, hypertension, hypothermia	Mechanical ventilation, doxapram	1	Survival

[7]	24	M	N/R	Conscious disturbance, diaphoresis, nausea/vomiting, premature ventricular beats, headache	Monitoring	0.5	Survival

[7]	29	M	N/R	Bradycardia, cerebral hemorrhage, conscious disturbance, diaphoresis, ectopic ventricular beat, headache, nausea/vomiting	Evacuation of cerebral hemorrhage atropine	3	Survival

[8]	53	M	N/R	Hypertension, pulmonary edema	Mechanical ventilation	4	Survival

[8]	40	M	N/R	Diaphoresis, headache, hypertension, nausea/vomiting, pulmonary edema	O_2_ via mask, doxapram	1	Survival

[8]	32	M	N/R	Bradycardia, conscious disturbance, diaphoresis, hypertension, pulmonary edema	Nicardipine, mechanical ventilation	2	Survival

[9]	40	M	140	Bradycardia, hypertension, pulmonary edema	Atropine, nicardipine, diuretics, doxapram, O_2_ via mask	2	Survival

[10]	21	F	150	Bradycardia, kidney dysfunction, liver dysfunction, miosis, pulmonary edema, QT elongation	Renal replacement therapy, mechanical ventilation, siverastat, urinastatin	3	Survival

[11]	23	M	150	Kidney dysfunction, liver dysfunction, nausea/vomiting	Renal replacement therapy, diuretics	30	Survival

Current case	22	F	160	Bradycardia, hypercoagulopathy, kidney dysfunction, liver dysfunction, nausea/vomiting	Renal replacement therapy, Alprostadil	72	Survival

N/R, not recorded.
